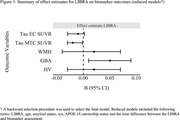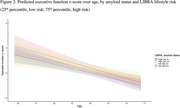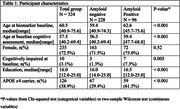# Associations of late mid‐life lifestyle with brain and cognitive changes in individuals on the AD vs. non‐AD continuum

**DOI:** 10.1002/alz.087218

**Published:** 2025-01-09

**Authors:** Julie Elisabeth Oomens, Karly Alex Cody, Lianlian Du, Erin M. Jonaitis, Rachel L Studer, Caitlin Artz, Madilynn Wintlend, Sebastian Köhler, Pieter Jelle Visser, Stephanie J. B. Vos, Rebecca E. Langhough, Willemijn J. Jansen, Sterling C. Johnson

**Affiliations:** ^1^ Alzheimer Center Limburg, School for Mental Health and Neuroscience, Maastricht University, Maastricht Netherlands; ^2^ School of Medicine and Public Health, University of Wisconsin‐Madison, Madison, WI USA; ^3^ Stanford University School of Medicine, Stanford, CA USA; ^4^ Department of Biostatistics and Medical Informatics, School of Medicine and Public Health, University of Wisconsin‐Madison, Madison, WI USA; ^5^ Wisconsin Alzheimer’s Institute, University of Wisconsin‐Madison School of Medicine and Public Health, Madison, WI USA; ^6^ Department of Medicine, University of Wisconsin‐Madison School of Medicine and Public Health, Madison, WI USA; ^7^ Wisconsin Alzheimer’s Disease Research Center, University of Wisconsin‐Madison School of Medicine and Public Health, Madison, WI USA; ^8^ Alzheimer Center and Department of Neurology, Amsterdam Neuroscience Campus, VU University Medical Center, Amsterdam Netherlands; ^9^ Geriatric Research Education and Clinical Center, William S. Middleton Memorial Veterans Hospital, Madison, WI USA; ^10^ Wisconsin Alzheimer’s Institute, University of Wisconsin School of Medicine and Public Health, Madison, WI USA

## Abstract

**Background:**

To aid development of prevention strategies, we investigated whether a composite measure of late‐midlife lifestyle health was associated with (1) change in brain tau burden, vascular burden and neurodegeneration and (2) cognitive trajectories when accounting for these brain changes.

**Method:**

We included 324 individuals from the Wisconsin Registry for Alzheimer’s Prevention. Late‐midlife lifestyle was assessed using the Lifestyle for Brain Health (LIBRA) score, encompassing 12 risk‐and protective factors for cognitive decline and dementia. Biomarker outcomes included regional tau PET burden in the entorhinal cortex (EC) and a meta‐temporal composite (MTC; mean(SD) FU time 2.4(0.6) years) as well z‐scores for WMH (FU time 2.6(0.77) years), hippocampal volume and global brain atrophy (HV; GBA; FU time 5.7(3.1) years). Cognitive outcomes included Preclinical Alzheimer’s Cognitive Composite (PACC‐3) and memory and executive domain performance (FU time 6.6(3.5) years; z‐scored). Amyloid status was determined based on global cortical average PiB DVR (cut‐off>1.16). We used linear mixed effect models to examine whether LIBRA modified age‐related tau, WMH, neurodegeneration and cognitive trajectories across amyloid positive and negative participants. Models for cognitive outcomes also accounted for MTC tau burden and GBA and their interactions with age.

**Result:**

Participant characteristics are given in table 1. LIBRA did not modify age‐related biomarker trajectories, independent of amyloid status. Reduced models indicated LIBRA main effects on MTC SUVR and GBA, such that higher late‐midlife LIBRA scores, indicative of worse brain‐lifestyle, were associated with lower (better) MTC SUVR values and higher GBA z‐scores (indicating more atrophy; b=‐0.02, p=0.017 and b=0.05, p=0.018 respectively; Figure 1). LIBRA was differentially associated with executive function trajectories in amyloid positive vs. negative participants (p interaction 0.019; Figure 2) while there was no association of LIBRA with PACC3 or memory trajectories.

**Conclusion:**

Lifestyle health was differentially associated with executive function trajectories, but did not modify age‐related biomarker, PACC3 or memory trajectories. We saw a counterintuitive association of lifestyle health with tau burden, and lifestyle health was associated with GBA. Improving our understanding of the contribution of lifestyle factors to brain and cognitive changes in the pre‐dementia stage will aid the development of prevention strategies.